# Immunology of RNA-based vaccines: The critical interplay between inflammation and expression

**DOI:** 10.1016/j.ymthe.2025.09.011

**Published:** 2025-09-08

**Authors:** John S. Tregoning, Ziyin Wang, Saranya Sridhar, Robin J. Shattock, Frank DeRosa

**Affiliations:** 1 Department of Infectious Disease, Imperial, London SW7 2AZ, UK; 2 Sanofi, Reading, Earley, UK; 3 Sanofi, Waltham, MA, USA

**Keywords:** RNA vaccine, inflammation, expression, formulation, delivery, safety, manufacture

## Abstract

Since its use during the COVID-19 pandemic, mRNA has emerged as a leading candidate vaccine platform for pandemic infections. A critical difference between RNA-encoded antigen and protein vaccines is that RNA-based vaccines require the antigen to be translated in the body, adding an important variable. Much of the research focus in the field has been on ways to increase expression, but inflammation plays a critical role. The vaccine delivered is a combination of the RNA and the formulation, so both elements need to be considered. Formulated RNA can act as a form of adjuvant but can also activate cellular pathways that inhibit expression. Expression and inflammation are interlinked, but independent—a deeper understanding of the quality and quantity of immune induction will help to develop more efficient RNA vaccines. Here, we discuss factors that shape responses to RNA-based vaccines. These include the composition of the vaccine (the use of modified RNA bases, whether self-replicating or traditional mRNA and, critically, the formulation) and the type of cells that take up and translate the RNA. We then consider challenges presented by current generation RNA vaccines including clinical impact and how improved immunological understanding can inform the development of improved RNA vaccine platforms.

## Graphical abstract

**Figure:**
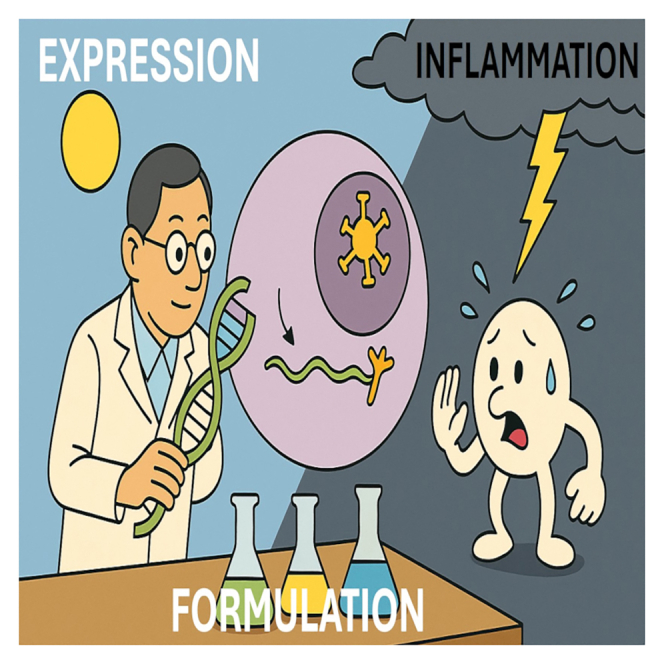

## Teaser

Given the explosion of RNA vaccines in the last 5 years, this review explores gaps in knowledge and what is needed to fill these. Tregoning and colleagues focus on the immune response to RNA vaccines, specifically on the interplay of expression and inflammation and how formulation affects this.

## Introduction

The licensure of RNA vaccines for COVID-19 was a major milestone for this technology. It not only validated the RNA-based vaccine platform for reduction of disease following viral infections, but further increased the interest and investment in the application of mRNA for other prophylactic and therapeutic indications such as protein replacement therapy, gene editing approaches, and personalized cancer vaccines.^1^ A number of unanswered questions relating to the immunology of RNA vaccines remain. The focus of this review is the interplay of expression and inflammation in the induction of adaptive immune responses and associated reactogenicity.

Vaccines provide protection by training the immune system to recognize pathogens prior to infection, laying down immune memory.^2^ The effectors of this immune memory (called the adaptive response) are T cells, and antibodies produced by B cells. T cells can be divided into CD4 helper cells, which orchestrate the response and CD8 killer cells, which target infected cells. While some vaccines target polysaccharides, most target proteins (in the case of RNA vaccines, these are encoded by the mRNA). The protein targeted by the vaccine is called the antigen and the specific regions within it are called epitopes. When the vaccine is introduced into the body (normally by injection), the material is detected by innate immune cells, these communicate with other cells through the release of secreted messengers in a process called inflammation. Inflammation can lead to responses felt by the vaccinated individual including local symptoms such as swelling and pain, and systemic ones such as headache and fever; these responses are often described as reactogenicity.^3^

The immune response to vaccines, including RNA-based vaccines, can be defined in three phases: sensing by innate immune mechanisms, bridging by innate cells (shuttling and presenting antigen to adaptive immune cells), and the establishment of adaptive memory. However, a major difference between RNA and protein vaccine platforms is the requirement for endogenous expression of the encoded antigen, and how the immune response affects that expression. The innate immune system forms a major defense mechanism against viral infection and can inhibit expression from exogenous RNA through the induction of type I interferon (IFN) and the expression of interferon stimulated genes (ISGs). Viruses have evolved multiple ways to ensure their RNA can hijack host cells and be expressed. RNA vaccines have also achieved this, through packaging the RNA in delivery systems, such as lipid nanoparticles (LNPs), which successfully transfect multiple cell types and produce the desired protein. While the respective protein (antigen) production is desirable, these types of vaccines also induce local and systemic inflammation. Both the RNA payload and components of the formulation (particularly when LNPs are used) can act as pathogen-associated molecular patterns (PAMPs), which contribute strongly toward reactogenicity.^4^

Here, we examine immune factors that contribute to the success or failure of RNA vaccines. We initially explore the nature of the innate and adaptive immune responses elicited by RNA vaccines, before examining two critical variables that affect these responses—the composition of the RNA vaccine and location of antigen expression. Finally, we evaluate how our insights into RNA vaccine-induced immune responses can be applied to future RNA vaccine development and discuss current knowledge gaps including how inflammation affects reactogenicity.

### Immune responses to RNA vaccines

#### Innate immune response to RNA vaccines

A wide range of factors affect innate immune sensing of exogenously administered RNA. These include the formulation, the route of administration, the type of cell transfected, the intracellular location after uptake, and the structure, sequence, and quality of the RNA. A critical consideration is that the innate response to the vaccine is a response to multiple components acting in concert, both the RNA and the formulation in which it is delivered.

Some of the best-characterized sensors of unmodified single-stranded mRNA and its degradative products are Toll-like receptors 7 and 8 (TLR7/TLR8),^5^ which predominantly detect endosomal RNA. However, for LNP-formulated-modified mRNA, one study has identified that antibody responses induced by BNT162b2 were TLR independent.^6^ Suggesting that rather than detection in the endosome, RNA is detected by cytoplasmic sensors such as retinoic acid-inducible gene I (RIG-I)-like receptors, including RIG-I and MDA5, which activate the signaling protein MAVS.^7^^,^^8^ RIG-I recognizes a range of motifs in viral RNA,^9^ including 5′ tri- and di-phosphates.^10^ Most *in vitro* transcription (IVT) of RNA is performed using polymerase derived from bacteriophage T7, resulting in a 5′ triphosphate end (uncapped), which RIG-I recognizes as foreign.^10^ The resulting RNA can be further modified through enzymatic means to produce a fully capped mRNA, facilitating ribosome recognition and protein production, while minimizing unwanted innate immune stimulation through host pattern recognition receptors (PRRs). Among other functions, the incorporation of a eukaryotic 5′ Cap (Cap1 or Cap2) reduces recognition by host PRR and enhances translation.^11^ Alternative capping approaches, such as co-transcriptional capping via tri-nucleotide cap analogs, can also result in a successful endogenous 5′ cap species, which therefore reduces innate recognition.

In a Nobel-prize winning breakthrough, Kariko et al. demonstrated that incorporation of N1-methylpseudourine (m1Ψ) into RNA prevents the activation of TLR7, TLR8 and other innate immune sensors, including protein kinase R (PKR) and oligoadenylate synthetases (OAS).^12^ This leads to a reduction in the type I IFN response, thus allowing for better translation of mRNA *in vitro* and *in vivo*. Other modifications have also been proposed such as 5-methylcytidine,^7^ and there is a substantial catalog of potential modifications of all three aspects the phosphate backbone, ribose and the base from the siRNA field.^13^

The conformation of RNA also has an effect on immune recognition, as RNA in the unusual Z form can be sensed by the Z-RNA-binding protein1 (ZBP1).^14^ Uridine depletion has been demonstrated to result in better performance, presumably due to reduced TLR activation.^15^ Depending on nucleotide composition, other proteins also bind RNA, including FUS, CNBP, TIA-1, and PCBP1 which can affect immune recognition.^16^ In addition to the desired mRNA, unwanted impurities generated during the IVT reaction, such as double-stranded RNA (dsRNA) or prematurely aborted short RNAs, can also trigger the innate immune system (TLR3 and TLR7/8, RIG-I, respectively). The presence of dsRNA can be controlled through optimization of the production process (alternative polymerases, reaction conditions) and/or the purification process.^17^^,^^18^^,^^19^ dsRNA is an important PAMP and removing it reduces innate stimulation; we have observed a slight increase in antibody responses when additional purification steps were included, possibly because of reduced inflammation.^20^

Formulation also plays a critical role in the innate response. LNPs themselves have been shown to be inflammatory, inducing a wide range of cytokines and chemokines, and have even been used as adjuvants for protein vaccines.^21^^,^^22^ While studying LNP alone can inform us about their role, it is worth noting that LNP is ineffective as a vaccine on its own and the effect of both the mRNA and the LNP carrier system working together is critical for inducing the cytokine response necessary for an adaptive immune response to the encoded antigen, particularly the type I IFN response.^23^ Furthermore, the presence of RNA significantly influences the pH dependence of lipid ionization,^24^ and therefore may affect the behavior of the LNP in the cell.

**Figure 1:**
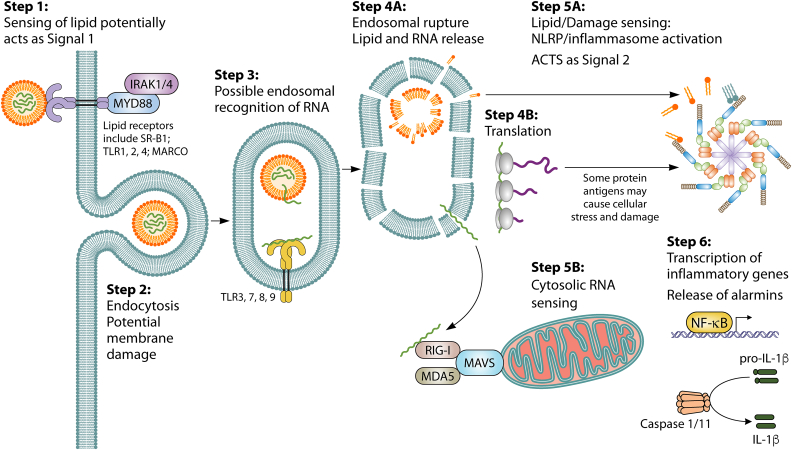
Sensing of LNP-RNA by cells A generalized response to unmodified RNA in an LNP. Changing formulation or type of RNA will alter outcome, as will encoded antigen. Step 1: LNP is sensed by the cell—this can act as signal 1, increasing transcription of inflammatory genes. Step 2: LNP taken up by endocytosis. Step 3: endosomal sensing of RNA (varies on RNA type). Step 4A: endosomal rupture—size of pores defined by ionizable lipid, which affects downstream effect. Step 4B: RNA is translated—some antigen cause stress to the cell. Step 5: RNA and LNP components sensed by cytosolic PRR. Step 6: downstream response—release of inflammatory mediators.

The mechanism by which LNP-RNA induces an inflammatory response has yet to be fully elucidated.^4^ But we propose the following as a generalized description, this will vary based on the LNP composition and the type of RNA (Figure 1). LNP may be sensed extracellularly, acting as signal one as a response to the lipid components. Lipids are sensed by a range of receptors including CR3, MARCO, SR-B1, and TLR1, 2, and 6—this has been observed in the context of *Mycobacterium tuberculosis*.^25^ Whether any of these interact with LNP needs further investigation, but a recent study has suggested that when MC3 is used as the ionizable lipid in the LNP it can interact with the TLR4/MyD88 pathway,^26^ not all ionizable lipids necessarily behave the same. In MyD88^−/−^ mice the T_FH_ and germinal center (GC) B cell responses the LNP-RNA vaccination were partially blunted.^27^ Another study showed that reactogenicity to empty LNP was reduced in Myd88^−/−^ mice.^26^ To enter the cell, the LNP is engulfed within endosomes; the mechanism of endosomal escape still needs further evaluation.^28^ Endosomal TLR can sense RNA, but the LNP type will influence whether the RNA interacts with these sensors, especially if the endosome ruptures before the TLR are present. The ionizable lipid is typically designed with pH sensitivities to fuse with the endosomal membrane and trigger escape, causing membrane damage.^29^ This releases endosomal membrane components that can stimulate the inflammasome,^30^ and RNA which activates cytosolic PRR such as RIG-I. When NLRP3 (the inflammasome sensor) was blocked with the inhibitor MCC950, the IL-1β response following RNA-LNP immunization was abolished.^30^ RNA-LNP sensing results in cell death if sufficient damage is done to the membrane or caspases are triggered.^31^ All of these factors then work in concert leading to inflammation and the recruitment and activation of immune cells.

Muscles, the main injection site, contain a large network of blood vessels that allow the recruitment of different types of immune cells, such as infiltrating dendritic cells, inflammatory monocytes and neutrophils. Immunization with RNA-LNP can lead to the activation of these antigen-presenting cells (APCs).^32^ This is either through the release of cytokines from cells that take up the RNA-LNP, or more directly if APCs take up the RNA-LNP itself. For example, a single intramuscular immunization with the COVID-19 vaccine BNT162b2 in mice activates DCs, monocytes, and macrophages in the draining lymph nodes, lungs, and spleen.^6^ This may be direct activation at the injection site, or from the LNP draining to the lymph node and acting there. RNA vaccination may also lead to innate imprinting (or trained innate immunity); this is where previous exposure to an immunostimulatory agent affects future innate responses.^33^ Following a second immunization, injection site APCs produce higher levels of types I and II IFN in the serum,^34^ although this effect is transient. The increased cytokine response is accompanied by a greater frequency of CD14^+^CD16^+^ inflammatory monocytes and an increased expression of ISG in these monocytes. Interestingly, a higher frequency of systemic side effects such as fatigue, headache, fever, and arthralgia were observed after the second vaccine dose.^35^ These data might reflect a connection between the induction of systemic reactions after secondary immunization in humans or in animal models following the mRNA-LNP vaccine and long-term functional changes in innate immune cells. Results from a recent study support this hypothesis. Long-term effects induced by BNT162b2 vaccination were observed in both transcriptional changes and cytokine production capacity when cells from immunized individuals were restimulated *ex vivo*.^36^ However, prior RNA exposure can boost or suppress responses, in a mouse model, prior exposure to an RNA vaccine had modest non-specific dampening effects on a secondary vaccination^37^; the gap between administrations may be important. And in PBMC collected from children after RNA vaccination, there were altered responses to stimulation *ex vivo*, with SARS-related antigens having elevated responses and bacterial antigens inducing lower responses comparing before and 21 days after vaccination.^38^

#### Role of inflammation in RNA vaccines

RNA vaccine-induced inflammation is critical in the outcome of immunization (both for efficacy and reactogenicity), the effector molecules for this are cytokines. One cytokine type that needs particular consideration is type I IFN, especially in the context of T cell responses. We and others have observed lower CD8^+^ T cell responses when IFNAR is blocked with antibodies during mRNA vaccine administration.^20^^,^^39^ IFNα has been used as an adjuvant for peptide vaccines to increase CD8^+^ T cell responses, suggesting an overlap in mechanism between protein and RNA vaccines.^40^ However, another study has observed reduced T cell responses in *IFNAR*^−/−^ knockout mice immunized with unmodified mRNA,^41^ and we have observed increased T cell responses to unmodified self-amplifying RNA (saRNA) vaccination in *IFNAR*^−/−^ and *MAVS*^−/−^ knockout mice^8^; although the mechanism of this remains unclear. These differences may be a function of the delivery route, when RNA was delivered subcutaneously, *IFNAR*^−/−^ had enhanced T cell responses, yet when delivered intravenously (i.v.), responses were lower in *IFNAR*^−/−^ mice.^42^ A recent study observed a role for a type I IFN-IL-27 axis in the induction of CD8 T cell responses.^43^

Type I IFN triggers a cascade of genes that inhibit translation, suppressing the amount of the antigen produced by the mRNA vaccine. Exogenous RNA can induce the expression of a range of ISGs including protein kinase R (PKR), RNAseL and OAS.^44^ In a recent publication, we compared the transcriptomic signature in injected mouse muscle, assessing the incorporation of m1Ψ, as well as the combination of m1Ψ, Cap1, and dsRNA purification. Exchanging uridine with m1Ψ and removing dsRNA significantly reduced the expression of ISGs including *RnaseL*, which has an RNA degradation role.^20^ One area that requires further research is the mechanism by which individual ISGs interact with RNA vaccine materials and affect expression/immunogenicity.

**Figure 2:**
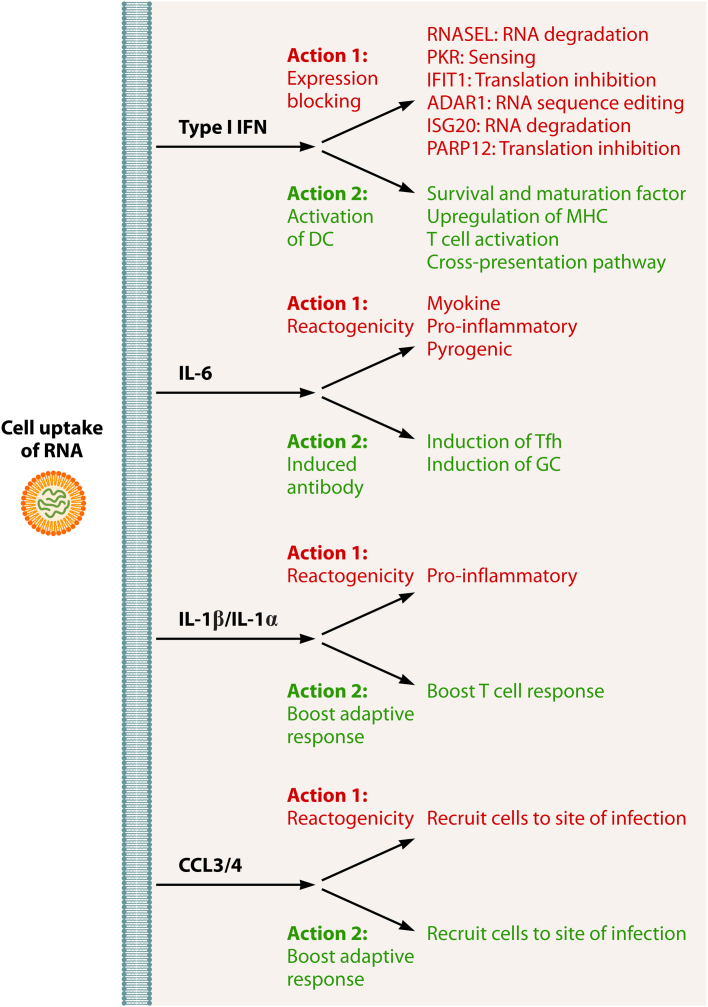
Cytokines are pleiotropic Cytokines induced by RNA vaccines have both beneficial and harmful effects. Getting the balance right is critical for an adaptive immune response without excess reactogenicity.

Type I IFN is not the only influential cytokine in the response to RNA vaccines. For saRNA, we have observed a correlation between formulations that induce greater systemic cytokine responses and high levels of antibody, in particular CXCL10, IL-6, IFN-γ, IL-5, and TNF.^8^ A similar pattern of cytokines was observed in another mouse model of vaccination.^45^ It is of note that patients with inflammatory bowel disease who are treated with anti-TNF (infliximab) had lower antibody titers than patients who were treated with an antibody targeting an integrin.^46^^,^^47^ In clinical studies, the levels of IFN-γ and CXCL10 correlated with antibody responses after the second^48^ and third immunizations^49^; interestingly, these studies also identified a correlation between antibody responses and the level of IL-15. The mechanism by which these cytokines boost the antibody response to RNA vaccines needs to be explored. One action might be through the induction of T follicular helper cells and GC B cells—and IL-6 has been shown to play a role in this,^27^ but further dissection is required; there is likely some redundancy between cytokines. An important consideration is the overlap between the cytokines needed to induce an adaptive immune response and those that trigger reactogenicity—for example, IL-6 is a myokine and associated with inflammation and fever (Figure 2). IL-6 and type I IFN are not alone in having a prozone effect—most vaccine-induced cytokines act in a prozone with enough needed to engage the adaptive immune system, but not too much to shut down expression of the RNA or induce unwanted side effects.

#### Adaptive immunity to RNA vaccines

Although the innate immune system is critical for inducing vaccine responses, it is predominantly vaccine-induced adaptive immunity that protects against infection and severe disease. With regard to the adaptive immune response, for most RNA vaccines targeted at viruses, the focus has been on inducing antibody-mediated protection, specifically on eliciting neutralizing antibodies that bind to and prevent viral entry. There was a significant correlation between antigen-specific antibody levels and serious COVID-19 outcomes. The antibody response to first-generation mRNA vaccines was acutely high, but protection from infection waned rapidly.^50^ This antibody waning has been associated with reduced effectiveness over time. In March 2021, protection against symptomatic infection was 91% for Comirnaty and 92% for Spikevax (the COVID-19 RNA vaccines from Pfizer/BioNTech and Moderna, respectively). However, protection decreased drastically after only 5 months to 50% and 62%, respectively.^51^ The neutralizing antibody response against the spike protein of five major SARS-CoV-2 variants in health care workers vaccinated with SARS-CoV-2 mRNA vaccines declined markedly from 1 to 6 months after the second mRNA vaccine dose.^52^ While this waning of effectiveness may be due to a reduced antibody titer, viral drift will also have contributed. The rapid mutation rate of SARS-CoV-2 meant that first-generation mRNA-based COVID-19 vaccines were insufficient in sustaining widespread protection as new variants emerged.^53^ There has also been a reported failure to establish SARS-CoV-2-specific plasma cells in bone marrow after mRNA vaccination.^54^ Some people have argued that waning antibodies may somehow potentiate infection, but there is no evidence for antibody-dependent enhancement in human coronavirus infection.^55^^,^^56^

In pre-clinical mouse and non-human primate models, mRNA vaccines encoding influenza HA trigger T_FH_ cell responses.^57^ Interestingly, studies in mice show stronger T_FH_ and GC B cell responses following vaccination with m1Ψ-containing mRNA than unmodified when formulated in an LNP.^58^ It remains unclear how m1Ψ promotes stronger GC reactions than unmodified mRNA, but this could be because protein translated from modified mRNA is generally expressed at a higher level and, for a longer duration getting more antigen to the lymph node.^32^ Alternatively, the adjuvant effect of the formulation may influence the induction of GC. Differences were seen between infection and immunization, mRNA vaccination with spike antigen led to a strong GC reaction, whereas SARS-CoV-2 infection led to a predominantly extrafollicular response.^59^

In addition to antibodies, T cells play a role in RNA vaccine-induced protective immunity against future infections. It has been argued that T cells can provide more durable responses against viral pathogens, especially those undergoing antigen drift.^60^ Human T cell responses following the COVID-19 mRNA vaccine rollout were observed in the PITCH (Protective Immunity from T Cells in Healthcare workers) cohort.^61^ Sustained T cell responses from 1 to 10 weeks after the first dose were observed,^62^ contrasting with the waning of T cells others have reported after natural infection.^63^ Dosing interval appears to impact the quality of the T cell response, with longer dosing intervals leading to a CD4^+^-dominated phenotype with marked IL-2 production.^64^ Interestingly, the antigen-specific CD8^+^ T cell response was higher following SARS-CoV-2 vaccination compared with infection.^65^ It is also worth noting that only peripheral T cell responses were analyzed in many of the human clinical studies. One study that examined the human airways found little evidence of lung mucosal T cells following RNA vaccination, compared with those induced by infection.^66^ If resident memory T cells (Trm) do perform a protective function, then approaches to induce them through RNA vaccination need refining. One recently proposed approach to boost Trm is the inclusion of the NKT agonist α-galactosylceramide with LNP-formulated RNA, which increased liver Trm and protected against malaria challenge.^67^ Likewise, the addition of cGAMP to LNP-formulated HA encoding mRNA increased Trm in the lung.^68^ But both studies were in pre-clinical models, so further translational validation is required.

Getting a balanced, durable, protective response with RNA vaccines remains a challenge. While a relatively short-lived, antibody-dominated response was appropriate for an outbreak of a virus with known sensitivity to neutralizing antibody (SARS-CoV-2), there may be a need for a qualitatively different response for other pathogens or applications. In particular, RNA-based cancer vaccines (where much of the original RNA vaccine research originated) need to induce a strong and lasting cytotoxic CD8^+^ T cell response necessitating a different quality of response or different induction pathways.

In the context of inflammation, one factor that will influence this response is pre-existing, low-grade chronic inflammation. This can be observed in the elderly, people with obesity, and patients with autoimmune diseases. People with immune-mediated inflammatory diseases had lower antibody responses and these declined faster^69^; however, it should be noted that many of these individuals were on antibodies blocking B or T cell function. Obesity is also associated with chronic inflammation; different studies have observed different impacts on the vaccine response in people with high body mass index (BMI). One study observed a marked lower antibody response in individuals with severe obesity (BMI > 40)^70^ another study observed no effect in individuals with BMI > 30.^71^ Age also influences baseline inflammation and has been shown to compromise anti-S titers to an mRNA booster vaccination.^72^ Interestingly, the likelihood of severe reactogenicity was lower in older age groups.^73^

Ultimately, the landscape of how RNA vaccines interact with the immune system is extremely complex. When taken in the context of the vast heterogeneity of human vaccinees, an empiric, trial-and-error approach may, to some degree, help identify the best options. There is probably a “goldilocks” (ideal) zone where inflammation is strong enough to induce an adaptive immune response, but not strong enough to shut down expression or affect tolerability. Further studies of immunological mechanisms, both in the pre-clinical and experimental setting should help to tease apart the interplay of factors and help shape future developments. It is worth noting that many studies to dissect the immune response have been performed in mice. While mice allow for interventional, mechanistic studies, the mouse model reflects many, but not all aspects of the human response. One area of difference that has been highlighted is in the differential role of IL-1ra in different species.^30^ It has been argued that, based on a dose/bodyweight calculation, mice are less sensitive to LPS,^74^ and an equivalent argument was made for RNA vaccines in mice when equivalent final doses are used (50 μg per mouse). But it should be noted that the response across inbred strains of mice varies and that some strains may reflect aspects of the human response better than others, depending on the question being asked. There are also challenges with selecting the best *in vitro* approaches for screening formulations; often expression is evaluated, but in the absence of cytokine release. Organoids can add complexity to individual cell models but will lack the dynamics and kinetics of *in vivo* systems. Likewise, there are limitations with current generation AI simulations as a complete replacement for broader understanding. Ultimately, combining data from different approaches is the best way to address complex immunological questions, with *in vivo* models playing an important part.

There is scope to manipulate responses to RNA vaccines to optimize their efficacy. One of the most hotly explored is formulation.

### Composition: What the RNA vaccine is made of

#### Formulations

While naked mRNA can cross into cells, the efficiency is extremely low—RNA is a large, hydrophilic, negatively charged macromolecule and hence does not cross the hydrophobic lipid cell membrane. Furthermore, eukaryotic cells have evolved a large number of ways to protect themselves against the threat of foreign RNA—including nucleases and RNA-sensing PRRs.^4^ For these reasons, to induce optimum immune responses, RNA needs to be formulated appropriately.^75^ But formulation performs a broader function than simply getting the RNA across the cell membrane. It can influence the cell types to be targeted, where it ends up in the cell, and critically the type of inflammatory response the vaccine induces. The underpinning principle of RNA formulation is to both protect the RNA from nuclease degradation and overcome the charge and polarity of the nucleic acid. This can be done with positively charged macromolecules, lipids, or a combination of the two. An extraordinary range of approaches have been proposed, with much inspiration drawn from experience with the delivery of smaller therapeutic RNA molecules, such as antisense oligonucleotides or siRNA.^76^ A review from 2018 cataloged the reported immune effects related to different formulations and observed that 61% of lipid-based nanoparticles had some effect, whereas 65% of polymer-based nanoparticles did not report an effect.^77^

#### LNPs

The two licensed mRNA vaccines for COVID-19 were formulated in LNPs, although they had subtly different formulations.^78^ In addition to these, one of the licensed saRNA vaccines also used an LNP (ARCT-154). LNPs tend to be composed of four elements: an ionizable lipid, a helper lipid, cholesterol, and a PEGylated lipid. The cholesterol plays a role in stabilizing the LNP bilayer but is presumed immunologically inert. The other three components play an important role in immune responses to the LNP.

Polyethylene glycol (PEG) is a commonly used, generally recognized as safe, family of stabilizing excipients that are included in a very broad range of drugs, household products, and cosmetics.^24^ For RNA vaccines, PEG increases the stability of the formulation—preventing LNPs from aggregation and increasing its half-life in the circulation. PEG also reduces the interaction with opsonin, thereby potentially reducing uptake by phagocytic cells.^79^ However, PEG can induce an antibody response, probably through a T cell-independent mechanism.^80^ This has been reported for PEGylated liposomes as far back as 2000, with an observed acceleration in blood clearance.^81^ While PEG by itself may not be directly immunogenic, when it is coupled to larger molecules it acts as a hapten, inducing anti-PEG IgM antibodies.^82^ In mice the induction of anti-PEG IgM is driven by Marginal Zone B cells in the spleen.^83^ A range of factors influence the induction of anti-PEG antibodies including PEG chain length, hydrophobicity, and molecular weight; a higher dose of phospholipid has been suggested to be protective by inducing anergy.^84^

Increased levels of PEG-specific antibodies have also been observed after RNA vaccination.^85^ Anti-PEG antibodies can cause two effects accelerated clearance of the vaccine and a pseudo-allergic reaction. The accelerated clearance from the blood stream and lymphatics occurs via increased uptake into phagocytes.^85^ Acute allergic reactions to PEG-containing LNPs in COVID-19 RNA vaccines have been documented and estimated at a rate of 7.91 cases per million (based on 41,000,000 vaccinations).^86^ These reactions are not classical allergy; a recent study has concluded that anti-PEG IgE is not the predominant mechanism for anaphylaxis post-mRNA vaccination.^87^ Side effects from PEGylated compounds are commonly referred to as complement activation-related pseudo-allergic reactions and have been described for other PEGylated liposomes—for example, liposomal doxorubicin (Doxil),^88^ and are most likely driven by the IgM. PEGylated nanoparticles also directly activate the complement cascade, which has been proposed to be due to their close resemblance to viral particles.^89^

Both the ionizable and helper lipid components play an important role in modulating the properties of the LNP and a wide range of natural and synthetic molecules have been evaluated.^90^ In pre-clinical studies, altering either the helper lipid or the ionizable lipid both altered the cytokine release profile after immunization.^91^ But of the two, the ionizable lipid is believed to be more dominant as an immunomodulator.^92^ This may be attributed to their role in endosomal escape, leading to the release of PAMPs within the cell,^93^ in turn triggering the inflammasome.^31^ Increasing the proportion of ionizable lipid in the LNP increased antibody titers following vaccination.^27^ Separately, the inclusion of ionizable lipid resulted in an observed increase in leukocyte recruitment following ID injection.^94^ Different profiles are seen with different ionizable lipids, for example, SM-102 (used in Moderna’s mRNA-1273 vaccine) induces more IL-1β than MC3^30^ and, in another study, similar levels of activation were seen with a proprietary lipid, ALC315 (used in BioNTech’s BNT162b2 vaccine) and SM-102.^95^ Modifying the amine head group has a greater impact than changing the acrylate tail on the innate immune response to ionizable lipids.^96^ This effect was proposed to be driven by the nature of the interaction with the MD2 binding cavity in TLR4 and interference with lipid-raft formation. The molecular structure of ionizable lipids is a critical factor; cone-shaped geometry characterized by a compact hydrophilic head group (binding the RNA) and a bulky hydrophobic tail leads to the endosomal destabilization.^24^ Engineering the ionizable lipid so it is biodegradable can reduce the level of inflammation, while retaining the overall immunogenicity.^97^

The ratio of number of RNA copies per LNP is not uniform, a recent study has suggested that the mean is two copies but this can go up to six, with a presence of 40%–80% empty LNPs depending on conditions.^98^ This may have an influence on the downstream immune response.

#### Other formulations

A diverse range of cationic polymers have been proposed to deliver RNA. As well as synthetic polymers, cationic polypeptides have been trialed. The first protective mRNA vaccine used protamine, which is an arginine-rich molecule derived from fish sperm.^99^ One of the most commonly used (pre-clinical) polymers is polyethyleneimine (PEI), which is highly effective for *in vitro* and *in vivo* delivery but has not been translated into clinical success. Challenges with PEI are commonly attributed to its lack of degradability and its cytotoxicity; the cytotoxicity is related to PEI charge and structure.^100^ PEI may also have inflammatory properties and has been proposed as a protein adjuvant.^101^ Modification of the polymer backbone can alter the profile of the induced immune response. Alternative polymers which afford better biodegradability and higher tolerability (pre-clinically) include poly(β-amino esters) (PBAE), poly(amido-amine) (PAMAM), and poly(CBA-*co*-4-amino-1-butanol (pABOL), to name a few.^102^^,^^103^^,^^104^^,^^105^^,^^106^ The clinical development of polymers has been somewhat eclipsed by the success of LNPs in the COVID-19 mRNA vaccines, but further research is ongoing, as there may be qualitative benefits, particularly for protein replacement therapy, if polymers can increase RNA expression but reduce inflammation.

Cationic nanoemulsions (CNEs) are stable emulsions of oil and water. Where LNPs form a shell of lipid that encapsulates the RNA, CNE are droplets of lipid with the RNA on the outside. Similar oil and water emulsions are used as adjuvants for protein vaccines, for example, MF59.^107^ The same MF59 emulsion has also been used to deliver mRNA pre-clinically.^108^ Modifying the properties of the CNE can alter the biodistribution of the formulated vaccine, with a view to focus it in the muscle or lymph node, rather than more systemic distribution.^109^ For example, Gemcovac (the saRNA vaccine from HDT Bio) licensed in India, uses a form of the LION CNE formulation.^110^ Whether changing the distribution alters the quality of the response or reactogenicity of the vaccine needs further investigation.

Altering the distribution of the vaccine through its formulation raises an important question about direct targeting of specific cells—and whether this is something that should be sought. An assumption has been made that targeting APCs, because of their direct interaction with adaptive immune cells, would be preferential. This can be achieved by using specific lipids, DC-specific ligands (such as mannose), or the conjugation of cell-targeting mAbs to LNP surfaces.^111^^,^^112^ A substantial body of research has investigated tailoring formulations to target specific organs, for example, the inclusion of a fifth component to LNP to focus the response,^113^ with the suggestion that altering the charge of the LNP,^114^ or amino acid tail length,^115^ can redirect the targeting. However, as alluded to above, understanding which cells express the RNA-encoded antigen and the impact this has on the immune response would benefit from further exploration facilitating specific targeting of the most relevant cells.

#### RNA sequence, structure, and chemistry

Another area for fine-tuning is the role of the RNA as its own adjuvant. RNA is a PAMP, especially if the vaccine has high levels of dsRNA or has features associated with viral RNA such as lacking eukaryotic cap structures. Whether this adjuvant effect is beneficial or not needs further investigation. As with the LNP, the RNA will not be delivered independently, so it needs to be considered in the round—as RNA-LNP. But in the same way that changes can be made to the LNP element of the vaccine, changes can also be made to the RNA element.

Changing the sequence of the RNA itself can have an impact on RNA sensing as well as potency.^116^ For a gene the length of hemagglutinin, there are theoretically 10^200^ different possible sequences, all of which encode the same protein structure. One approach is codon optimization, which can employ many different algorithms to improve expression.^116^ A traditional approach is to select for codons with higher frequency use in humans. For example, the 6 codons that encode leucine vary in frequency from 40.3/thousand (CUG) to 7/thousand (UUA), although it should be noted that frequency changes with species. The relative frequency of a codon correlates to the abundance of its cognate tRNA, thereby possibly affecting protein assembly speed. The mRNA sequence can also impact the secondary structure, for example, by inducing more stable hairpins, and depending upon where in the transcript these occur, they can alter expression.^117^

One of the most important advances for RNA vaccines was the demonstration that altering nucleosides altered the innate immune response.^12^ When comparing modified mRNA head-to-head with unmodified RNA, significantly different transcriptomic and inflammatory signatures can be observed^20^ Comirnaty and Spikevax both incorporated N1-methylpseudouridine (m1Ψ). One question arises about the impact of modification on the translated protein, with a suggestion that m1Ψ can cause +1 ribosomal frameshifting, leading to a T cell response to the alternative peptides.^118^ And while it has been assumed that the modification is necessary, an unmodified mRNA vaccine from Curevac (CVnCoV) also demonstrated protective efficacy in phase 2B/3 trials,^119^ although this was not taken forward for licensure. There are also multiple ongoing trials using unmodified RNA for cancer vaccines where more IFN-I may be beneficial (NCT05938387 targeting glioblastoma, NCT05938387 targeting advanced non-small cell lung cancer, and NCT03289962 targeting metastatic tumors).

An alternative to messenger RNA is saRNA. This vaccine platform is derived from alphaviruses (Table 1), retaining a replicon, but removing the packaging elements.^122^ The first study using an alphavirus replicon was published in 1989, using Sindbis virus,^123^ but other alphaviruses are now used, most commonly Venezuelan equine encephalitis virus (VEEV). These vaccines interact with slightly different pathways to mRNA because they need to replicate. saRNA has a dsRNA step in its replication (although this can be concealed from the cell’s intrinsic sensing machinery in spherules—membrane-derived ultrastructures).^124^ However, the production of dsRNA can induce caspase-dependent apoptotic death.^125^ Some studies have explored the incorporation of alternative nucleotides into saRNA; for example, 5-methylcytidine to reduce unwanted innate immune stimulation.^7^ saRNA may also cause stress to the cell through the replication of the RNA; we have recently observed that transgene accounted for nearly 10% of total RNA reads in transfected cells.^126^

**Table 1:** RNA vaccine modalities

Vaccine type	Messenger (mRNA)	Self-amplifying (saRNA)	Circular (circRNA)
Licensed product	3 licensed products: COVID-19: Comirnaty (BioNTech) Spikevax (Moderna) RSV: mRESVIA (Moderna)	2 licensed products COVID-19: ARCT-154 (Arcturus) Gemcovac-OM (Gennova)	none
Base chemistry	licensed products 1-methylpseudouridine wild-type bases have been used in other clinical trials	wild-type bases (mostly) 5-methylcytosine has been trialled^120^	wild-type bases
5′ structure	AG clean cap (cap 1)	AU clean cap (cap 1)	no cap (circular) cap-independent translation (e.g., internal ribosome entry site)
Formulation	licensed products use LNP. various other approaches tried pre-clinically	ARCT-154 (LNP) Gemcovac (CNE) various other approaches tried pre-clinically	pre-clinical models have used LNP^121^ various approaches possible
Replicates	no	yes	no
Structure	mRNA alone	alphavirus (VEEV, SeFV) replicon with antigen inserted	circular
Advantages	smaller RNA molecule—potentially easier to manufacture/clean up	replicates *in situ*—potential for substantial dose reduction	more stable, prolonged expression

Another RNA platform is circular RNA, which are single-stranded RNAs that have been closed into a circle with no 5′ or 3′ end. First discovered in plant viruses in the 1970s, they were subsequently identified in eukaryotic cells. They are generated as linear precursors and then circularized. Because they lack a 5′ end, they require an internal ribosome entry site for translation initiation. These types of RNA have been tested as vaccines in pre-clinical models, for example, targeting mpox virus,^121^ and are proposed to be more stable and have a longer expression profile than linear mRNA.^127^ They have yet to be tested in clinical studies and hold some challenges for manufacturing at large scale that need to be overcome.

There probably is not a “one-size-fits-all” composition for RNA vaccines. Different applications may need different formulations. Improved understanding about how formulated RNA induces an immune response and the quality of that response is required, as is improved understanding about the type of immunity required to protect against infection. Formulation has a significant impact on where the RNA is delivered and where the antigen is made, driving the subsequent adaptive response.

#### Location: Where the antigen is made

RNA vaccines have to be translated *in situ*. There is still a lack of clarity regarding which cells the vaccine-derived RNA is translated and, critically, what impact the “geography” of antigen expression has on downstream responses.^128^^,^^129^ Much more research is needed to understand biodistribution and expression and whether the conclusions can be generalized or are specific to the model and formulation used.

#### Organ level expression

*In vivo* imaging in mice can provide a whole-organism view of transfection and how delivery route influences it, but there are limits of interpretation to larger animals/humans. There are three predominant systemic delivery routes for RNA—intramuscular, subcutaneous/intradermal, and intravenous. The route of delivery of mRNA-LNP drug products has a significant impact on the biodistribution of protein expression.^130^ A recent whole-mouse imaging study has explored LNP-RNA distribution across a range of immunization routes at a single-cell level^131^; this approach may enable future studies to fine-tune formulations.

The most common route for immunization is intramuscular (i.m.) delivery, which leads to more localized uptake of RNA and expression of the antigen, but still leads to broader biodistribution. In pre-clinical toxicology studies supporting the licensure of Comirnaty (Pfizer/BioNTech), ^3^H-labeled lipids were used to follow distribution of i.m.-delivered LNP-RNA. Most ^3^H was detected in the injection site, with the next highest levels in plasma and liver, but all tissues had low but detectable levels of radioactivity.^132^ Using a branched DNA assay (a chemiluminescent approach for the detection of nucleic acid that uses signal amplification, as opposed to PCR which uses target amplification),^133^ vaccine-derived mRNA was widely detectable across multiple tissues. However, the majority (by two orders of magnitude) was located in the muscle and local draining lymph nodes.^134^ A similar pattern was seen following RNA vaccination in non-human primates by PET-CT with the majority of drug product detected at the injection site, while a portion drained to the lymph node.^135^ When the longevity of detection was compared in rats, RNA was detectable 60 days after injection in the muscle, lymph node, and spleen.^136^ These studies indicate that the majority of i.m.-injected RNA LNPs stay in the muscle or draining lymph nodes, which may be important for limiting reactogenicity. Reducing systemic exposure completely could improve systemic reactogenicity and tolerability.

A similar localized pattern was seen after intradermal (i.d.) or subcutaneous (s.c.) delivery, where the injection site was the main site of RNA detection, although there was some fraction detected in the spleen.^134^ s.c. immunization led to localized luciferase expression at the site of injection, with some expression in the draining lymph nodes.^137^ A human clinical trial compared different routes of administration for effect on immunogenicity and reactogenicity.^138^ The study compared i.m. delivery with two forms of i.d. dosing—using a standard needle or with a specially designed short needle. They observed non-inferiority between groups when the amount of antigen was matched; but a secondary endpoint testing whether i.d. delivery could reduce the dose required to achieve the same sero-conversion level was not met.^138^

It has been widely reported that i.v. delivered RNA LNPs predominantly distribute to the liver and spleen, primarily through passive targeting.^130^^,^^139^^,^^140^ The liver has been a target of interest with mRNA, not only for liver-related diseases, but also serving as a protein production depot for mRNA-derived secreted protein therapies for distal organs such as kidney and heart.^141^^,^^142^^,^^143^ Recent studies have shown that through controlling the lipid composition and surface charge, one can selectively enrich in various organs (liver, spleen, lung).^144^ Whether RNA vaccine material reaches the spleen directly or is indirectly shuttled by APCs is unclear. The spleen has a predominance of immune cells, while the liver is predominantly composed of hepatocytes. Hepatocytes are highly metabolically active and can produce high levels of proteins so are seen as a promising target for therapeutics.^145^ However, there is a high turnover of liver cells, so the expression may be more transient. As a mass vaccination route, i.v. is unlikely to be used in humans due to the complexities of delivery. It could have a role for more bespoke RNA therapies, such as protein replacement or siRNA therapy, where expression level is more important than immune response.

### Zooming in: Cellular level expression

Whole-organism studies are helpful for indicating broad patterns of uptake and expression, but do not directly inform which cells are making the antigen (or responding to it). Alternative approaches are needed to identify specific antigen-expressing cells, including the use of RNA-delivered CRE recombinase to remove *LoxP* sites from around a fluorescent protein, effectively fate mapping the cell that took up the RNA. The most common model is the Ai9 mouse (or B6.Cg-*Gt(ROSA)26Sor*^*tm9(CAG-tdTomato)Hze*^/J); which has a red tdTomato transgene under Lox repression.^146^ Using this approach, expression in immune cells has been confirmed.^137^ i.v. delivery of RNA led to different expression patterns in different organs. In the lung and spleen, expression was predominantly observed in macrophages, whereas in the liver, expression was seen in macrophages, endothelial cells, and hepatocytes.^147^ In another study, following i.v. immunization, flow cytometric analysis of cells in the spleen found tdTomato expression in a range of cells, with the largest proportion in DCs.^148^ Using a related model with GFP under the Lox restriction rather than tdTomato, expression was explored in the lungs after retro-orbital injection of RNA. This indicated more expression in endothelial cells compared with blood cells (57%:36% proportionately).^149^ A combination approach has also been used, labeling both the LNP with a lipid dye (Atto655) and using RNA expressing a fluorescent protein (mCitrine). In this study uptake and expression of the RNA was observed in neutrophils and macrophages.^32^ Following vaccination of mice, single-cell RNA-seq on lymph nodes revealed spike mRNA in a range of immune cells, but predominantly in macrophages, APCs, and neutrophils.^6^ Whether or not RNA-delivered antigen is expressed in muscle cells following injection is unclear. One study demonstrated β-galactosidase (β-Gal) expression following mRNA administration in muscle cells,^108^ but another study reported vaccine-derived RNA in muscle cells, but not expression.^150^ The same study also observed expression in APCs in an NHP system. However, a functional study delivering CRISPR-gRNA targeting a muscular gene (dystrophin) demonstrated gene editing in mouse muscle cells following i.m. delivery.^151^ As of yet, no broadly generalizable patterns have emerged from these studies and further investigation is required.

Most work conducted to understand the cellular target of RNA has been performed in mouse models. Assessing its translatability to humans using *in vitro* or *ex vivo* studies with human cells or tissue explants is important. It is possible to transfect human skin and perform whole-tissue imaging.^152^ In another study, when skin explants were dissected for flow cytometry following immunization with RNA encoding GFP, antigen was detected in a range of different cell types, including epithelial cells, monocytes, and DCs.^153^ Spike antigen was detected in sera in the pg/mL range following mRNA vaccination of human volunteers; peaking around days 3–5 after immunization, preceding the antibody response.^154^ Interestingly, the antigen was not detectable after the second dose; similar levels were reported in the plasma in another study.^155^ Vaccine-derived RNA was detectable in the axillary (draining) lymph nodes of recently vaccinated, deceased patients up to 30 days after vaccination, but not in deceased patients who had received the vaccine more than 30 days before their death. Moreover, spike antigen was detectable in fine needle biopsies of human lymph nodes for a similar time period after injection with the spike-encoding mRNA vaccine.^156^ Using much more sensitive methodologies spike has been detected between 69 and 187 days after immunization,^157^ and potentially far longer with fM amounts found up to 709 days after immunization.^158^ Whether these very low amounts of protein have a functional impact needs further investigation. One issue for further evaluation is the expression profile of saRNA vaccines which may be different from mRNA vaccines.

Although these studies demonstrate that RNA vaccines can enter and be expressed in a range of cell types, they do not necessarily address the functional impact of the location of expression on the immune response. Does changing the route of immunization change the cell type expressing the antigen and therefore the quality of the immune response? If APCs take up RNA, is the antigen produced directly in the cell and presented to T cells?

**Figure 3:**
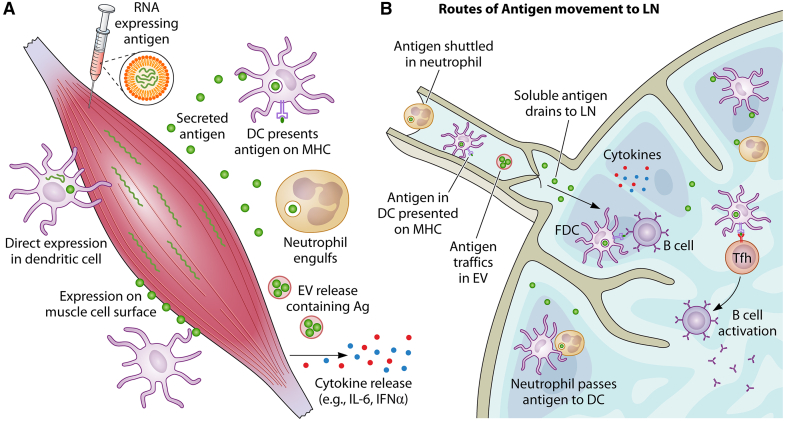
Location, location, location (A) Types of antigen expression and release from the i.m. injection site. (B) Possible mechanisms of antigen trafficking to lymph node.

Another question relates to how B cells interact with expressed antigen (Figure 3). Spike antigen was detected in the serum following RNA vaccination for COVID-19 in animal models,^6^ and human volunteers,^154^ so it may track to lymph nodes via drainage. Alternatively, spike protein has been detected in exosomes, which shuttle antigen between the vaccination site and the lymph node.^159^ One further possible mechanism is that neutrophils shuttle antigen to the lymph node; although one study has shown neutrophil depletion has limited effect.^160^ Another aspect that needs further exploration in the context of antigen distribution is the impact of soluble compared with membrane bound antigen. In a mouse model of saRNA-expressed HIV antigen, membrane bound antigen induced humoral responses with a faster kinetic profile.^161^

While the understanding of expression, both at an organ and a cellular level, and the exact influence of formulation upon them is incomplete, they remain hot areas of research and improved understanding will help address challenges associated with RNA vaccines.

### The future and challenges for RNA vaccines

Despite the rise of mRNA as a platform for use in both therapeutic and vaccine modalities, there are still significant challenges.^162^ The mRNA vaccines for COVID-19 have worked well in a pandemic setting and in subsequent annual vaccination campaigns. News of the approval of mRESVIA, a respiratory syncytial virus (RSV) vaccine developed by Moderna,^163^ means that mRNA-based vaccines for two different pathogens have received health authority approval so far (Table 2). There are many other antigens being targeted by RNA-based vaccines, at various phases of clinical trials (Table 2). Although not all of these will demonstrate efficacy, it reflects the investment in the platform, especially the number of different companies exploring them.

**Table 2:** Pipeline of phase 2, 3, and licensed RNA vaccines

Antigen	RNA type/formulation	Company	Ref/NCT
Licensed			
SARS-CoV-2 spike (Wuhan variant) Commercial name: Comirnaty	modified mRNA/LNP	Pfizer/BioNTech	^35^
SARS-CoV-2 spike (Wuhan variant) Commercial name: Spikevax Updated to Omicron BA.1 (2022) 2024 recommendation JN.1	modified mRNA/LNP	Moderna	^164^
Monovalent spike Wuhan-Hu-1 Commercial name: ARCT-154	saRNA	Arcturus/CSL	^165^
RSV F surface glycoprotein Commercial name: mRESVIA	modified mRNA	Moderna	^166^; NCT05127434
SARS-CoV-2 spike (Omicron variant) Commercial name: Gemcovac-OM	saRNA/CNE	Gennova	^167^
Phase 3			
Quadrivalent influenza HA (H1, H3, B-Vic, B-Yam variants)	modified mRNA	Pfizer	NCT05540522
Norovirus VP1 from three genotypes: GII.4, GI.3, and GII.3	modified mRNA	Moderna	NCT06592794
CMV Antigen glycoprotein B (gB) and gH/gL/UL128/UL130/UL131A pentametric complex	modified mRNA	Moderna	^168^^,^^169^; NCT05085366
Combination with other vaccines			
RNA COVID + pneumococcal conjugate SARS-CoV-2 spike (Wuhan variant)	modified mRNA	Pfizer	^170^
RNA COVID + influenza proteins (IIV) RNA component: SARS-CoV-2 spike Protein component: quadrivalent influenza HA (H1, H3, B-Vic, B-Yam variants)	saRNA (ARCT-2303) or modified mRNA (BioNTech/Pfizer)	Arcturus; BioNTech; Moderna	Arcturus (NCT06279871); BioNTech (NCT06178991) ^171^; Moderna (NCT06097273)
Influenza HA quadrivalent	modified mRNA (mRNA-1010)	Moderna	Moderna (NCT05415462; NCT05566639) phase 1/2 results^172^
RNA COVID + HPV RNA component: SARS-CoV-2 spike (Omicron Variant)	modified mRNA	MSD	NCT05119855
Phase 2			
Recurrent pulmonary osteosarcoma	tumor RNA	University of Florida	NCT05660408
TB: multiple undisclosed antigens BNT164a1 and BNT164b1	modified mRNA	BioNTech	NCT05547464
Malaria (*P. falciparum*) Antigen PfCSP BNT165e	modified mRNA	BioNTech	NCT06069544
RSV F glycoprotein in older adults	modified mRNA	Sanofi	NCT06251024
Autologous neo-antigens	modified mRNA	National Cancer Institute (NCI)	NCT03480152
VZV/shingles Glycoprotein E (gE)	modified mRNA	Pfizer	NCT05703607
Prostate cancer	mRNA	Curevac	NCT00831467
RSV F surface glycoprotein In mothers and children	modified mRNA	Moderna	mothers (NCT06143046) children (NCT06097299)
Norovirus antigen not disclosed	modified mRNA	Moderna	NCT05992935
SARS-CoV-2 spike	saRNA	Elixirgen Therapeutics	NCT04863131 phase 1/2 data^173^

The following considerations remain for the mRNA platform.

#### Nature of the antigen

A key feature of the mRNA platform is its ability to be a “plug-and-play” technology that can incorporate different antigens. An important question is whether the inflammation following RNA vaccination is antigen agnostic, or whether the encoded antigen influences inflammation. Some encoded proteins cause cellular stress, for example, GFP can lead to oxidative stress in the cells,^174^ and other antigens may have similar effects.

The mRNA vaccines approved so far encode viral glycoproteins in a pre-fusion state and show high levels of short-term efficacy. The hemagglutinin of the influenza B viruses appears to be a more difficult target, with data from different developers suggesting a “class” effect with regard to the poor immunogenicity. A phase 3 trial by Moderna (NCT05415462) compared a quadrivalent influenza virus vaccine (mRNA-1010) with an inactivated split vaccine. In this study, non-inferiority was not met for either endpoints for the influenza B/Victoria- and B/Yamagata-lineage strains.^175^ Although the cause of this remains unclear, one hypothesis is that the antigen expressed from the mRNA vaccine construct *in vivo* may be different from the one that is expressed in a wild-type/recombinant system, with considerations about glycosylation, trimer formation, and the need or not to express on the cell surface or as a soluble antigen. How these variables affect inflammation also needs investigating.

Whether the mRNA platform will work with more complex antigens from bacterial, parasitic or fungal pathogens, which tend to be larger or have different glycosylation on their surface, remains to be studied. Pre-clinical efficacy has been demonstrated for some bacterial targets including plague,^176^ group A streptococcus,^177^ *Acinetobacter baumannii*,^178^ and *Pseudomonas aeruginosa*.^179^ Likewise, protection has been demonstrated against liver-stage malarial infections with an mRNA vaccine.^67^ RNA vaccines targeting other pathogens have now progressed to clinical trials, for example, with an ongoing phase 1 trial for the treatment of acne (NCT06316297), a phase 1/2 trial for *Chlamydia* (NCT06891417) and ongoing phase 2 trials for mRNA-based vaccines targeting malaria and tuberculosis.

One area where RNA vaccines may be particularly beneficial is in combining multiple different antigens to generate a syndromic vaccine that protects against pathogens that cause similar diseases. For example, a winter respiratory disease vaccine (RSV, hMPV, influenza, and COVID-19)^180^ or one to reduce antibiotic-resistant bacterial pathogens following hospitalization (targeting the ESKAPE pathogens). Some early steps have been taken. Moderna have recently finished a phase 3 trial (NCT06097273) of a COVID-Influenza combined mRNA vaccine and observed non-inferiority to an inactivated viral (influenza) vaccine combined with an mRNA (COVID-19) vaccine.^181^ Increasing the valency of this vaccine to include RSV and potentially other winter season respiratory infections would reduce the number of injection visits needed by those at risk, increasing uptake. A phase 1 trial is being undertaken to explore this (NCT05585632).

An important consideration is how many antigens can be incorporated in an RNA vaccine. There is likely to be a limit on the level of injected RNA that can be tolerated, which is estimated at around 100–200 μg,^182^ limiting the number of antigens that can be safely included before reactogenicity issues are encountered. There may be issues relating to immune dominance, or original antigenic sin leading to a focusing of immune responses on some but not all of the antigens—although this is not seen in multivalent protein or glycoconjugate vaccines. One pre-clinical study in mice used a 20-valent RNA vaccine against influenza and detected responses to all strains.^183^ Whether this result would translate into humans has not yet been tested as the complexity of natural exposure and human variation make such approaches more challenging.

In the context of combination vaccines, saRNA could prove beneficial, because less RNA is required.^184^ In mouse models, it is possible to use as little as 10 ng saRNA and still induce a protective immune response.^8^ In a recent study, human volunteers boosted with ARCT-154 had greater booster responses and slower decline than a BNT162b2 booster.^185^ The first generation of saRNA vaccines led to 80% seroconversion when priming responses against a neoantigen.^186^ However, they have also been shown to be effective as a booster dose. Further immunological investigation and optimization based on these findings is required to fully deliver the promise of the platform as a clinical vaccine.^187^^,^^188^

Understanding the limits and devising new strategies to expand the use of the mRNA platform across a broad range of antigens will be the next frontier for mRNA research. As discussed above, a key area of research is the interaction between the antigen sequence and components of the delivery vehicle to understand and modulate the mechanisms underlying this reactogenic response.

#### The balancing act between immunogenicity and reactogenicity

A feature of the first generation of mRNA COVID-19 vaccines was the high rate of side effects observed compared with some traditional vaccine platforms.^189^ This has also been observed with new mRNA influenza vaccines compared with seasonal influenza vaccines made with inactivated and recombinant technology.^172^ In the context of the pandemic and an effective vaccine, the benefit-risk consideration was positive.^190^^,^^191^ But reactogenicity may affect end user uptake—with a trade-off in the context of combination vaccines, where a single vaccination covers several pathogens, and where slightly higher reactogenicity might be preferable to multiple immunizations.

A key challenge for the next generation of mRNA vaccines is to reduce the reactogenicity while maintaining a potent level of durable immunity. A few observations confirm the complexity of understanding and balancing these levels. A study of mRNAs developed by Moderna showed that similar mRNA doses and the same LNP induce higher levels of side effects with influenza antigens compared with an RSV antigen or COVID-19 antigens.^166^^,^^172^ The mechanism behind this observation remains to be elucidated: it may be a function of the type of RNA, sequence of the antigen, the multivalent nature of influenza vaccines, historical exposure to antigen, or the translated protein.^192^

The next generation of mRNA-based vaccines will need to investigate new strategies to modulate innate immune sensing to reduce reactogenicity while maintaining immunogenicity. These strategies include additional or new base modifications to balance exogenous mRNA translation and innate immune stimulation, improved purification removing residual innate stimulating impurities, new cationic lipids, or the use of alternative delivery systems such as polymers, as well as identifying ways to reduce the total mRNA dose.

A further complication is that, while reactogenicity and immunogenicity tend to correlate, they do not necessarily predict efficacy. One study with 189 participants observed a low correlation between redness (female) or fever (male) and neutralizing titer^193^; another study with 38 volunteers found some correlation between adverse reactions and antibody titer (only in men).^194^ More research is required, but the early data suggest that reactogenicity could be reduced without affecting immunogenicity.

#### Potential safety aspects of RNA vaccines

As a relatively new technology, with an extremely wide uptake mRNA vaccines have come under intense scrutiny since their rollout during the COVID-19 pandemic. An important consideration for adverse events is the widespread uptake of the vaccine, as of June 2023, 535 million doses of BNT162b2 had been administered,^195^ with 49.6 million doses administered worldwide in a single day at the peak. In a study evaluating AESI in 183,559,462 doses of BNT162b2 and 36,178,442 doses of mRNA-1273 in 99,068,901 people, there was a significantly increased risk of myocarditis and pericarditis^196^; but the absolute number of cases was in the low 100s—with a denominator in the 100s of millions. These events are very rare. More generally, a recent preprint has described self-identified cases of post-vaccine syndrome, which covers a range of fatigue-like symptoms.^197^ A follow-up preprint evaluated immune responses in 35 individuals who self-identified with post-vaccine syndrome and observed subtly different T cell profiles in PBMC, whether these are mechanistically important needs further investigation.^158^ The authors state that: “We emphasize the critical task of discerning between meaningful results and random fluctuations in the data.”^158^ Given the enormous number of doses administered and the heterogeneity of human responses, that there are some adverse events in some recipients is not wildly surprising. This can be seen for any given thing consumed by humans; for example, a recent report showed that 21 children had acute adverse events following the consumption of something as innocuous as slushed ice drinks.^198^ Ultimately, a large, pan-European survey of BNT162b2 clearly demonstrated that the benefits outweighed the risks.^195^

However, there are a number of considerations relating to inflammation, expression, and localization that may affect the clinical and safety profile of RNA vaccines. Some of this will be driven by the manufacturing and formulation process. *In vitro* transcription can generate a number of potentially inflammatory biproducts, for example, dsRNA and abortive RNAs, these need to be removed to reduce additional immune responses.^199^ One area that has been highlighted by some groups as a possible risk is plasmid DNA contamination^200^; but the methodology in this study has been called into question by a follow-up preprint.^201^ It is also worth noting that the Therapeutic Goods Administration of Australia measured 212 batches of Comirnaty and Spikevax for DNA contamination and found levels below 10 ng/dose.^202^ Another important aspect is to consider the impact of biodistribution of the LNP-RNA and the expressed protein. As described earlier, there is some systemic distribution of the LNP and the expressed protein, and further investigation is required to evaluate the impact of this. A pre-clinical study in mice observed a short spike (4–6 h) in spike mRNA in fetal liver after a large volume (100 μL) i.m. dose into the mother.^203^ However, large meta-analysis (17,719,495 pregnant persons and 389 pregnant animals) observed no safety signal of mRNA vaccination in pregnancy.^204^ Further studies will help to determine the impact of the biodistribution of mRNA vaccines and redesigning formulations to better localize them will be an important approach.

#### Vaccines for specific populations

The first generation of mRNA vaccines were developed for adults and, in particular, deployed to protect older adults. COVID-19 vaccines were also deployed in pediatric populations although vaccine uptake has decreased over time. The clinical development of COVID-19 vaccines for the pediatric population involved a dose de-escalation in younger age groups. However, the reactogenicity profile of COVID-19 mRNA vaccines in younger adult populations has restricted the use of these vaccines in this population, so further risk-benefit calculations may need to be applied to these age groups. The next generation of mRNA vaccines will need to be re-designed, keeping different age groups in mind. There might be a requirement for further modulation of the mRNA vaccine to be able to ensure that vaccines are designed specifically for younger populations. One aspect of this is to increase the level of IgA induced by RNA vaccines, which can reduce transmission. The first-generation COVID-19 mRNA vaccines were very effective at reducing severe disease and death,^190^^,^^191^ but lost the ability to block transmission as the virus drifted and as anti-S titers dropped. Another avenue for development of next-generation mRNA vaccines is mucosally delivered mRNA vaccines. It is likely that the nature of RNA formulation delivered to mucosal sites (upper respiratory tract or gut) will need to be different from current parenterally administered vaccines. The balance between local reactogenicity and generation of local immune response will depend on the type of RNA sequence and LNP formulation used. Such a delivery route may help in the induction of local antibodies and resident memory T cells to limit transmission.

Another target population where the first generation of mRNA vaccines have not been particularly successful are immune-compromised hosts, in particular transplant patients and those on immunosuppressive chemotherapy.^205^ Also, as discussed, underlying inflammation can affect both immunogenicity and reactogenicity. Vaccines with greater potency and better durability will be needed to ensure effective protection in these populations, especially for those on immunomodulating drugs or monoclonal therapy.

## Conclusion

It is worth emphasizing the extraordinary success of mRNA vaccines during the COVID-19 pandemic and the transformative effect of that success on the field. Prior to 2020, there had been fewer than 10 phase 1 trials of mRNA vaccines (targeting HIV, lung cancer, melanoma, influenza virus, and rabies virus), with the first infectious disease mRNA vaccine trial happening as recently as 2017.^206^ The final selection of dose, composition, antigen, formulation, and regime for the vaccine was based on informed, but ultimately lucky, judgment. Now that the pandemic is over, more research is required to refine this powerful platform for wider use. Addressing where the antigen is expressed and the impact this has on the response, the role inflammation plays in the response and managing the balance between reactogenicity and immunogenicity are key areas where immunology can inform the development of the platform. There are also wider issues relating to manufacture and acceptability, which, while not directly determined by immunology, can be informed by it. The heterogeneity of the human response to any drug also needs considering; however, RNA vaccines, because of the manufacturing scale, enable more precision vaccinology approaches. This platform is now a critical weapon in the vaccinologist’s arsenal, especially for pandemics; how we use it going forward will depend on greater understanding of the interplay between inflammation and expression.

## Acknowledgments

The authors thank Daniel Gonçalves-Carneiro for helpful discussions. We thank Patrick Lane, ScEYEnce Studios for the image rendering. J.S.T. and R.J.S. are supported by funding from the 10.13039/501100000266EPSRC Vaccine Hub and the NIHR BRC.

## Author contributions

Conceptualization, J.S.T.; writing – original draft, J.S.T., Z.W., and S.S.; writing – review & editing, J.S.T., Z.W., S.S., R.J.S., and F.D.R.

## Declaration of interests

S.S. and F.D.R. are employees of Sanofi. J.S.T. has advised the Sanofi influenza vaccine program and Moderna. R.J.S. has patents relating to RNA vaccination.
